# Long-Term Domiciliary High-Flow Nasal Therapy in Patients with Bronchiectasis: A Preliminary Retrospective Observational Case-Control Study

**DOI:** 10.3390/jcm11247323

**Published:** 2022-12-09

**Authors:** Claudia Crimi, Santi Nolasco, Raffaele Campisi, Mattia Nigro, Pietro Impellizzeri, Andrea Cortegiani, Alberto Noto, Andrea Gramegna, Carlo Vancheri, Francesco Blasi, Nunzio Crimi, Stefano Aliberti, Annalisa Carlucci

**Affiliations:** 1 Department of Clinical and Experimental Medicine, University of Catania, 95123 Catania, Italy; 2 Respiratory Medicine Unit, Policlinico “G. Rodolico-San Marco” University Hospital, 95123 Catania, Italy; 3 Department of Biomedical Sciences, Humanitas University, 20072 Milan, Italy; 4 IRCCS Humanitas Research Hospital, Via Manzoni 56, Rozzano, 20089 Milan, Italy; 5 Department of Surgical, Oncological and Oral Science (Di.Chir.On.S.), University of Palermo, 90127 Palermo, Italy; 6 Department of Anesthesia, Intensive Care and Emergency, Policlinico Paolo Giaccone, University of Palermo, 90127 Palermo, Italy; 7 Department of Human Pathology of the Adult and Evolutive Age “Gaetano Barresi”, Division of Anesthesia and Intensive Care, University of Messina, Policlinico “G. Martino”, 98125 Messina, Italy; 8 Department of Pathophysiology and Transplantation, Università degli Studi di Milano, 20122 Milano, Italy; 9 Internal Medicine Department, Respiratory Unit and Adult Cystic Fibrosis Center, Fondazione IRCCS Cà Granda Ospedale Maggiore Policlinico Milano, 20122 Milano, Italy; 10 Respiratory Unit, IRCCS Humanitas Research Hospital, Via Manzoni 56, Rozzano, 20089 Milan, Italy; 11 Department of Medicine and Surgery, Università degli Studi dell’Insubria, 21100 Varese, Italy; 12 Pulmonary Rehabilitation Unit, Istituti Clinici Scientifici Maugeri, 27100 Pavia, Italy

**Keywords:** high-flow nasal cannula, high-flow nasal therapy, bronchiectasis, mucus, exacerbation, hospitalization

## Abstract

High-flow nasal therapy (HFNT) provides several pathophysiological benefits in chronic respiratory disorders. We aimed to evaluate the effectiveness of long-term HFNT in patients with bronchiectasis (BE). Methods: This is a retrospective bicentric case-control study of outpatients with BE on optimized medical treatment with a severe exacerbation requiring hospitalization in the previous year. Patients on long-term home HFNT (cases) and patients on optimized medical treatment alone (controls) were matched by age, sex, bronchiectasis severity index, and exacerbations in the previous year. Data on BE exacerbations, hospitalizations/year, mucus features, respiratory symptoms, and pulmonary function were collected. The primary outcome was the change from baseline in the exacerbation rates at 12 months between groups. Results: 20 patients in the HFNT group and 20 controls were included. A significant reduction in exacerbations [−1.9 (−2.8 to −0.9), *p* = 0.0005] and hospitalizations [−0.7 (−1.1 to −0.3), *p* = 0.0006] was found in the HFNT group vs controls. A slight improvement in pulmonary function [FEV_1_% +6,1% (+1% to +11.3%) (*p* = 0.0219), FVC% +4.6% (+0.8% to +8.3%) (*p* = 0.0188) and FEF_25–75_% +13.4 (+11 to +15.9) (*p* = 0.0189) was also found in the HFNT group compared to controls. Conclusions: In this preliminary study, long-term domiciliary HFNT improved the clinical course of patients with BE.

## 1. Introduction

Bronchiectasis (BE) is a chronic and heterogeneous respiratory disease characterized by abnormal and irreversible dilatation of the bronchi, impaired clearance of secretions, chronic cough, sputum production, and history of respiratory infections [1,2]. Once a neglected disease, BE prevalence alone or as a comorbid condition [3,4,5] has increased by nearly 40% over the last decade [6], and it is associated with poor quality of life, frequent exacerbations, hospitalizations, morbidity, mortality, and substantial economic burden [7]. Excessive airway mucus provides a nutrient-rich nidus for chronic bacterial infection and inflammation that evolves over time, causes airflow obstruction, and promotes further progressive and irreversible structural lung damage [1,2]. Moreover, mucus hypersecretion and associated mucus plugging is a major cause of morbidity and mortality in chronic inflammatory airway diseases [8,9,10]; therefore, personalized chest physiotherapy interventions are crucial to affect sputum viscosity and volume and facilitate expectoration [11,12].

High-flow nasal therapy (HFNT) is a noninvasive respiratory support that delivers heated and humidified gases, eventually blended with oxygen, through special nasal prongs. HFNT yields several beneficial physiological effects, including wash-out of anatomical dead space, humidification and improved mucociliary clearance [13], reduced inspiratory effort, and improved respiratory mechanics [14,15], representing a promising management strategy for patients with chronic respiratory diseases. Thus, HFNT is being increasingly used in acute care settings for the management of patients with acute respiratory failure of different origins [16,17,18,19,20,21] and in selected patients with mild-to-moderate acute respiratory failure due to chronic obstructive pulmonary disease (COPD) [22,23]. However, limited evidence supports its long-term domiciliary use, mainly focusing on patients with COPD [24,25,26,27]. Given the strong physiological rationale and the documented clinical benefits of HFNT in muco-obstructive chronic respiratory disease, long-term domiciliary HFNT could be considered a reasonable add-on treatment to optimized medical and respiratory physiotherapy for patients with BE [28].

In this study, we aimed at evaluating the effects of the long-term use of HFNT on exacerbations, hospitalization rate, pulmonary function, and patient-related outcome measures in patients with BE.

## 2. Materials and Methods

### 2.1. Study Design

In this observational, retrospective, case-control pilot study, patients with BE who were referred to two tertiary-level outpatient clinics at the Policlinico University Hospital, Catania, Italy, and the Policlinico Hospital, Milan, Italy, between September 2018 and October 2021 were enrolled. The study protocol was approved by the “Catania 1” Ethics Committee of the Policlinico University Hospital (Approval Number 176/2018/PO, Catania, Italy) and adhered to the Declaration of Helsinki.

### 2.2. Patient Population

Consecutive adult patients (≥18 years old) were enrolled if all of the following were present: (i) a diagnosis of radiologically (at least one lobe on chest high-resolution computed tomography) and clinically significant BE [1]; (ii) a clinical history consistent with chronic cough, sputum production most days of the week and/or frequent respiratory infections [1]; (iii) at least one severe exacerbation of BE (defined as an exacerbation requiring hospital admission) in the previous year; (iv) optimized medical maintenance therapy, respiratory physiotherapy and pulmonary rehabilitation (performed by a specialized respiratory physiotherapist), including technical support for airway clearance, according to the European Respiratory Society (ERS) guidelines [29]; (v) underwent HFNT during the night for at least 12 months. Patients with cystic fibrosis as well as those with traction BE were excluded. Patients with COPD were included only if a primary diagnosis of BE was established and COPD was present as a comorbidity. All of the included patients were referred to the outpatient clinic in Catania, where we started prescribing long-term domiciliary HFNT in patients with BE who had at least one severe exacerbation requiring hospitalization in the previous year as per local policy starting from 2018.

### 2.3. Control Population

Controls were adult patients (≥18 years old) with a diagnosis of radiologically and clinically significant non-cystic fibrosis BE with a clinical history consistent with chronic cough, sputum production most days of the week, and/or frequent respiratory infections and at least one severe exacerbation of BE (defined as an exacerbation requiring hospital admission) in the previous year despite optimized medical maintenance therapy, respiratory physiotherapy and pulmonary rehabilitation [1] and with at least 12 months follow-up. Controls were individually matched for age (±5 years), sex, bronchiectasis severity index (BSI), and number of exacerbations in the previous year (±2 years), in a 1:1 ratio, from a group of 725 patients followed in the outpatient clinic of Milan.

### 2.4. High-Flow Nasal Therapy

According to standard operating procedures, HFNT was initiated for all the enrolled patients using a dedicated device (myAirvo 2, Fisher & Paykel Healthcare, Auckland, New Zealand) initially set at a gas flow of 25 L/min [30] and titrated upward to patient comfort up to 40 L/min. The temperature was set at 34°C or 37°C, according to patient tolerance. If necessary, oxygen supplementation was added and FiO_2_ was adjusted to maintain SpO_2_ ≥ 92%. The size of the nasal cannula (Optiflow; Fisher & Paykel Healthcare, Auckland, New Zealand) was selected to occlude a patient’s nostril of about 2/3 of their size. Patients underwent daytime HFNT acclimatization and setting titration. Upon prescription, they were instructed to use the device for a minimum of 6 h a day, preferably at night. The average time of HFNT use was calculated by dividing the total number of hours registered on the device by the number of days that occurred from the home delivery of the device to 12 months. None of the patients were treated with HFNT prior to the study period.

### 2.5. Data Collection and Outcome Assessment

An established database of relevant variables was accessed for the analysis. Demographic characteristics (age, sex, body mass index) and clinically relevant parameters were collected at baseline [31], including smoking history, comorbidities and previous medical history (including number of exacerbations and hospitalizations during the last year), BE etiology, maintenance therapy, airway clearance and/or pulmonary rehabilitation, modified Medical Research Council (mMRC) dyspnea scale [32], sputum color, difficulty of mucus expectoration assessed through a Visual Analog Scale (VAS), quality of life assessed using the Saint George Respiratory Questionnaire (SGRQ) [33,34], pulmonary function, peripheral oxygen saturation (SpO_2_) at rest, alpha-1 antitrypsin, Bhalla score [35], BSI [36] and chronic microbial colonization.

The number of exacerbations and hospitalizations, mMRC dyspnea scale, sputum color, difficulty of mucus expectoration (VAS), total SGRQ score, pulmonary function tests, and resting SpO_2_ were assessed at the 12-month follow-up.

BE exacerbation was defined as a clinical diagnosis of the physician determining a change in BE treatment in the presence of deterioration in three or more of the following symptoms for at least 48 h: cough, sputum volume and/or consistency, sputum purulence, breathlessness and/or exercise tolerance, fatigue and/or malaise or hemoptysis [31].

To assess the degree of functional disability due to dyspnea, the mMRC dyspnea scale was used. It ranges from “0”, implying dyspnea only with strenuous exercise, to “4” indicative of dyspnea at rest [32].

Sputum was defined as mucous if its color was white to light yellow, mucopurulent if yellow to light green, and purulent if green or dark green [37]. Patients rated their difficulty of sputum expectoration before and after treatment using a 10 cm visual analog scale (VAS, 0 = extremely easy; 10 = extremely difficult) [38], which asked “how difficult was it to cough and expectorate sputum?”. 

Symptoms and quality of life were evaluated with the SGRQ, a questionnaire encompassing 76 total items grouped into 3 domains, with higher values representing worse respiratory symptoms and quality of life (range 0–100) [33,34].

Pulmonary function tests were performed according to the ERS/ATS guidelines [39]. Data on pre-bronchodilator forced expiratory volume in the first second (FEV_1_%), forced vital capacity (FVC%), and forced expiratory flow between 25% and 75% of FVC (FEF_25–75_%) were collected at baseline and after 12 months.

### 2.6. Bronchiectasis Severity Evaluation

The radiological severity of BE was evaluated with the Bhalla score [35]. The Bhalla score range goes from 0 to 25, with a lower score indicating more radiologically severe BE. This score was subdivided into mild (16–25), moderate (9–15), and severe (0–8). We also assessed the number of lobes involved and the degree of bronchial dilatation using the Reid classification [40]. The clinical severity of BE was evaluated according to the BSI [36]. BE were defined as mild (BSI score = 0–4), moderate (BSI score = 5–8), or severe (BSI score ≥9 points). Microbiological examination was performed on patients’ spontaneous early morning sputum samples. We considered the presence of chronic colonization if the same pathogen was found in at least two sputum cultures with a minimum of 3 months apart for 1 year with the patient in a stable state [41].

### 2.7. Outcomes

The primary outcome was the change from baseline in the rate of exacerbations at 12 months in BE patients treated with long-term home HFNT compared to standard of care.

Secondary outcomes measures were rate of hospitalizations, changes in mMRC dyspnea scale, difficulty of mucus expectoration (VAS), sputum features, quality of life (SGRQ), and pulmonary function (FEV_1_, FVC, FEF_25–75_), after 12 months before and after treatment initiation and in both HFNT and control groups. Furthermore, we explored the relationship between HFNT utilization time on outcomes. Moreover, we assessed the safety of HFNT evaluating the adverse events (intolerance, poor tolerance) reported in the HFNT cohort.

### 2.8. Statistical Analysis

Categorical variables are stated as numbers (*n*) and percentages (%). Continuous variables are expressed as the mean ± standard deviation (SD). HFNT settings are presented using median and interquartile range (IQR). Data normality distribution was checked using Q–Q plots. Fisher exact or McNemar tests were used for comparisons of categorical variables, when appropriate. Mixed-effect models were applied for both within and between groups comparisons of annual exacerbation rate and hospitalizations. Mean differences and 95% confidence intervals (95% CI) were assessed to evaluate treatment effects. Relative Risks were also assessed, and the 95% CI was calculated using the Koopman asymptotic score. For continuous secondary outcomes, differences between treatment groups were assessed using unpaired Student’s *t*-test or unpaired Wilcoxon rank-sum test according to data distribution. Linear regression analysis was developed to evaluate the association between the time of HFNT use and changes in exacerbations, hospitalization, functional and spirometric parameters. According to Gaussian data distribution, Pearson (*r*) or Spearman’s (*ρ*) rank correlation coefficients were calculated. All statistical tests were two-tailed, and *p*-values <0.05 were considered to indicate statistical significance. Statistical analyses and figures were generated using GraphPad Prism (version 9.3.0) (GraphPad Software, San Diego, CA, USA) and SPSS (version 26) (IBM Corporation, Armonk, NY, USA). No a priori sample size calculation was performed, due to the nature of the study.

## 3. Results

### 3.1. Baseline Patient Characteristics

**Figure 1:**
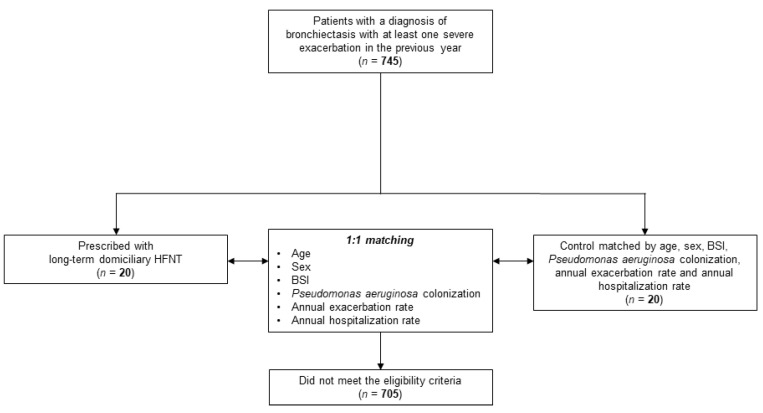
Study flow diagram. Abbreviations: HFNT, high-flow nasal therapy; BSI, bronchiectasis severity index.

Twenty patients who underwent long-term home HFNT were included and matched with 20 patients who did not undergo HFNT. A flow diagram of study participants is shown in Figure 1. An overview of the study cohort is shown in Table 1.

The majority of cases were female (28/40, 70%) with a mean age of 69.6 ± 9.1 years. Most common BE etiologies included post-infective (11/40, 27.5%) and associated with chronic obstructive pulmonary disease (10/40, 25%), while 25% of the patients had idiopathic BE. Patients’ clinical and radiological characteristics, disease severity, etiology, and sputum microbiology are reported in Table 2. The median (IQR) HFNT flow rate was 33 (25–40) L/min, median (IQR) temperature was 34 (34–37) °C, and median (IQR) fraction of inspired oxygen (FiO_2_) was 21% (21–35).

### 3.2. Primary Outcome

Comparisons before and after HFNT initiation and between cases and controls are reported in Table 3.

**Figure 2:**
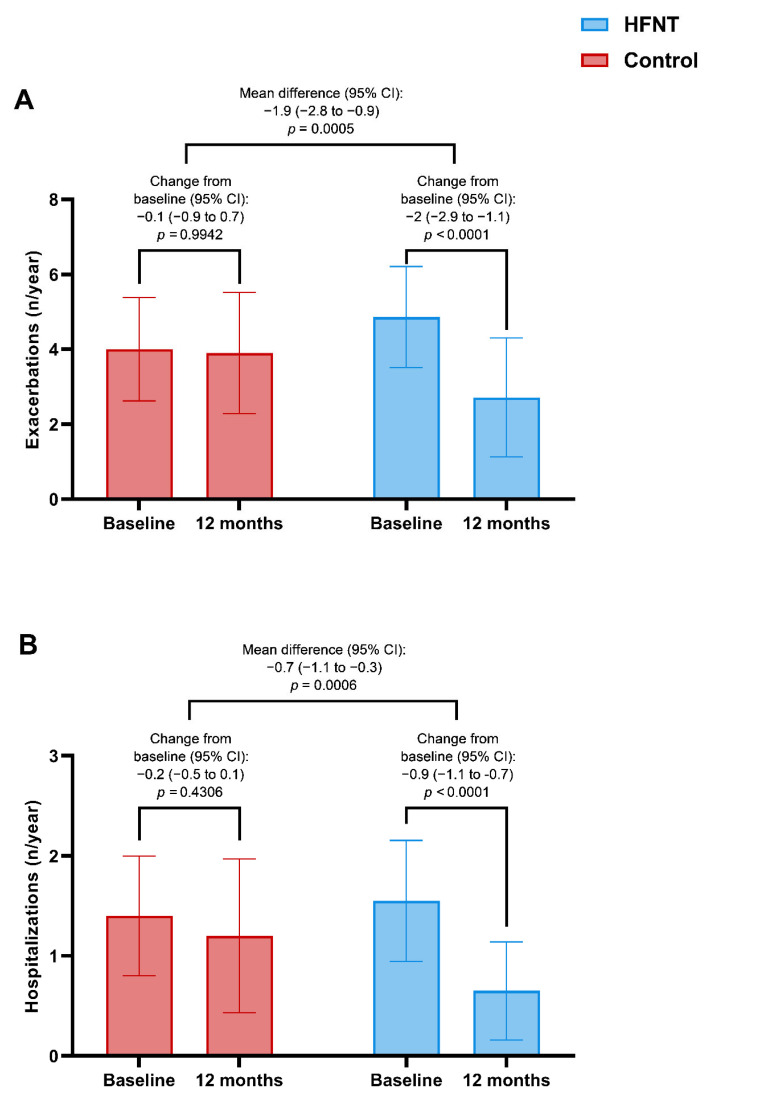
Exacerbations (**A**) and hospitalizations (**B**) in HFNT and control group at baseline and after 12 months. Abbreviations: HFNT, high-flow nasal therapy.

A significant improvement in the change of the exacerbations rate at 12 months was found in the HFNT group vs controls [−2 (−2.9 to −1.1) (*p* < 0.0001) vs −0.1 (−0.9 to 0.7) (*p* < 0.9942) respectively, with a between-group difference of −1.9 (−2.8 to −0.9) exacerbations/year, *p* = 0.0005] (Figure 2A).

### 3.3. Secondary Outcomes

The median annual hospitalization rate decreased from 1.6 (0.6) to 0.7 (0.5) (*p* < 0.0001) in the HFNT group with a between-group difference of −0.7 (−1.1 to −0.3) hospitalizations/year (*p* = 0.0006) (Figure 2B).

**Figure 3:**
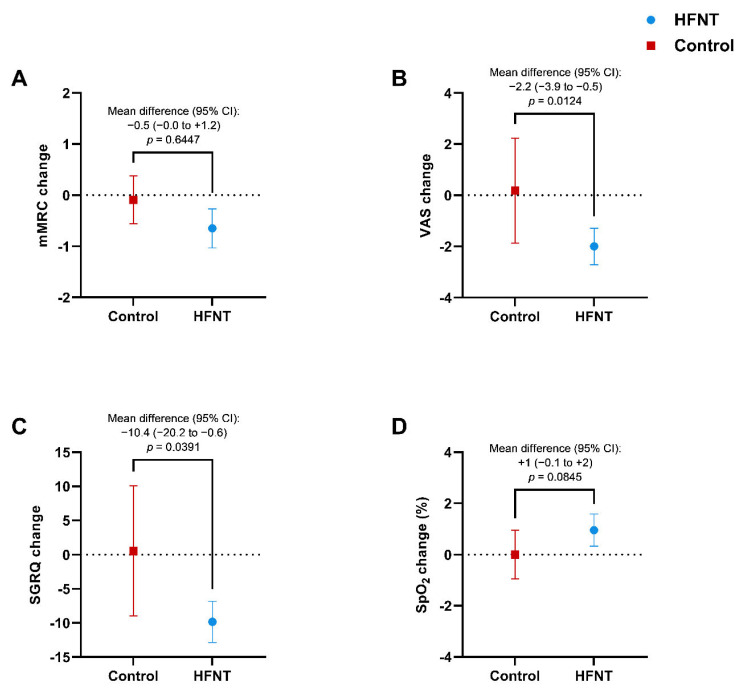
Changes in mMRC dyspnea scale (**A**), difficulty of expectoration (VAS) (**B**), SGRQ total score (**C**), and SpO_2_ at rest (**D**) in HFNT and control group at baseline and after 12 months. Data are expressed as mean (95% CI). Abbreviations: HFNT, high-flow nasal therapy; mMRC, modified medical research council; VAS, visual analog scale; SGRQ, Saint George Respiratory Questionnaire; SpO_2_, peripheral blood oxygen saturation; CI, confidence intervals.

The mMRC dyspnea scale score significantly improved by −0.6 points (−0.1 to −1.3) (*p* = 0.0129) after treatment in the HFNT group. However, no significant difference between cases and controls was found [−0.5 (−0.0 to +1.2), *p* = 0.6447] (Figure 3A) at 12 months. 

The VAS score for difficulty of mucus expectoration decreased by −2 (−2.7 to −1.3) points within the HFNT group (*p* = 0.0028) after 12 months, with a between-groups difference of −2.2 (−3.9 to −0.5) points (*p* = 0.0124) (Figure 3B). A statistically significant reduction of mucopurulent sputum in favor of mucoid expectoration [from 2/20 (10%) to 9/20 (45%), *p* = 0.0233] was found only in the HFNT group. The SGRQ score improved significantly in patients treated with HFNT, with a −9.9 decrease (−12.7 to −6.8) (*p* = 0.0012) (Figure 3C) resulting in a difference of −10.4 (−20.2 to −0.6) (*p* = 0.0391) vs control group. No difference in the SpO_2_ at rest was found within and between groups (Figure 3D). 

**Figure 4:**
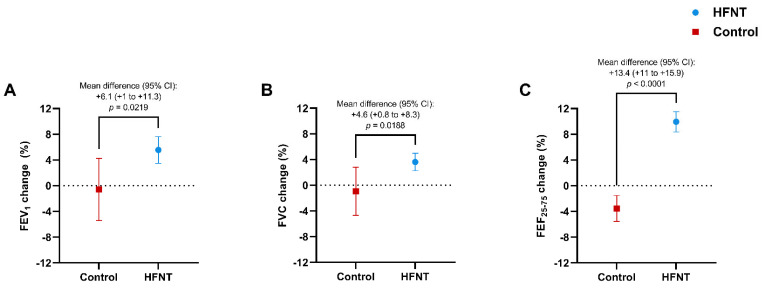
Changes in FEV_1_% (**A**), FVC% (**B**), and FEF_25–75_% (**C**) in HFNT and control group at baseline and after 12 months. Data are expressed as mean (95% CI). Abbreviations: HFNT, high-flow nasal therapy; FEV_1_, forced expiratory volume in the first second; FVC, forced vital capacity; FEF_25–75_, forced expiratory flow between 25% and 75% of FVC; CI, confidence intervals.

Pulmonary function tests showed a significant improvement in the HFNT group with an increase in pre-bronchodilator FEV_1_% of +6.1% (+1% to +11.3%) (*p* = 0.0219) (Figure 4A), FVC% of +4.6% (+0.8% to +8.3%) (*p* = 0.0188) (Figure 4B) and FEF_25–75_% of +13.4% (+11% to +15.9%) (*p* < 0.0001) (Figure 4C) vs controls.

### 3.4. Correlation between Time of High-Flow Nasal Therapy Use and Outcomes Changes

**Figure 5:**
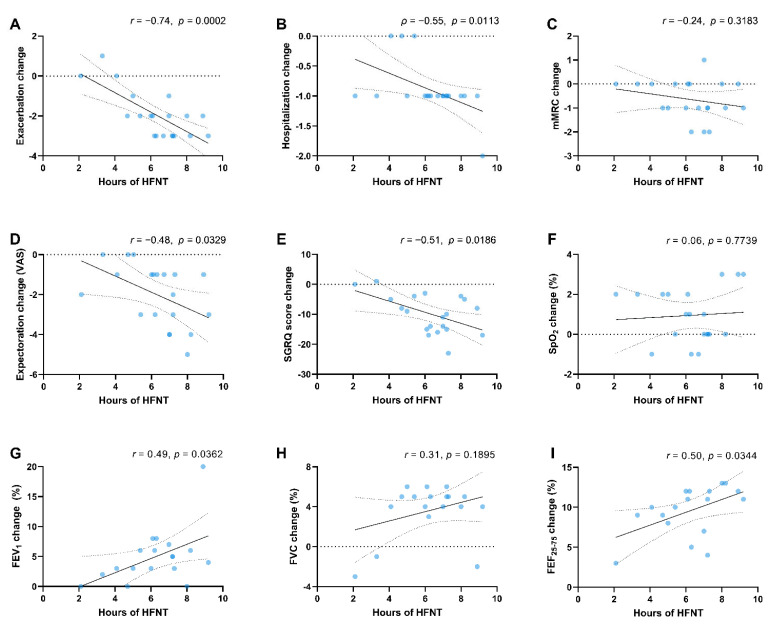
Scatter diagrams and regression lines (95% CI) on correlations between the amount of HFNT hours per day and changes in exacerbations (**A**) and hospitalizations (**B**) mMRC dyspnea scale (**C**), difficulty of expectoration (VAS) (**D**), SGRQ total score (**E**) and SpO_2_ at rest (**F**) FEV_1_% (**G**), FVC% (**H**), and FEF_25–75_% (**I**). *r* = Pearson coefficient, *ρ* = Spearman coefficient. Abbreviations: HFNT, high-flow nasal therapy; mMRC, modified medical research council; VAS, visual analog scale; SGRQ, Saint George Respiratory Questionnaire; SpO_2_, peripheral blood oxygen saturation; FEV_1_, forced expiratory volume in the first second; FVC, forced vital capacity; FEF_25–75_, forced expiratory flow between 25% and 75% of FVC.

The average time of use of HFNT was 6.3 ± 1.8 h per day (range: 2.1 to 9.2 h) with 14 patients out of 20 (70%) achieving a treatment adherence greater than 6 h. Correlation between HFNT use and annual exacerbation rate, hospitalizations, mMRC dyspnea scale score, difficulty of mucus expectoration, SGRQ score, SpO_2_, and pulmonary function are reported in Figure 5A,I.

### 3.5. Safety

No serious adverse events related to the device occurred throughout the 12 months. Only two patients stopped HFNT treatment prematurely: one because of poor tolerance and one due to personal reasons.

## 4. Discussion

This study showed that long-term home HFNT use on top of optimized medical and respiratory physiotherapy significantly reduces the annual exacerbations rate in patients with BE compared to standard of care. To the best of our knowledge, this is the first real-world study investigating the effectiveness of HFNT in a cohort of patients with non-cystic fibrosis BE with detailed baseline clinical and radiological characterization, including BSI, Bhalla score, and microbiological information.

BE disease activity is driven by a vicious vortex [42] of infection, inflammation, and tissue damage which further impairs host defenses and leads to disease progression [43,44]. Mucus hypersecretion and mucociliary clearance impairment reduce lung compliance, increase airway resistance and work of breathing, and are associated with increased risk of mucus plugging, impaired gas exchange, lung function decline, infections, and increased risk of hospitalization [45]. Despite the recognized importance of excess airway mucus in the mechanisms underlying BE formation, bacterial infection, and inflammation, to date, therapeutic options for airway mucus obstruction remain limited.

The delivery of warm and humidified air through the HFNT may optimize mucosal function, determine an increase in mucus water content, change the rheologic and viscoelastic properties of sputum, facilitate secretion removal, reduce airway, and potentially prevent bronchospasm [30,46,47] playing an important role in the treatment of patients with chronic muco-obstructive lung diseases [48,49,50]. HFNT providing gas at 100% relative humidity may preserve the effective mucociliary defense improving mucus clearance [51] in patients with alterations of the mucociliary clearance and airway obstructed by mucus plugs such as BE and COPD [52].

Although there is a solid physio-pathological rationale for using long-term HFNT in this patient population [30,53,54], there is a lack of evidence about its use in this clinical context [55]. Moreover, no consensus or guidelines exist on the domiciliary use of HFNT in patients with chronic respiratory disorders, and the few available reports focused mainly on patients with COPD [24,25,26,27]. A post hoc analysis of the Rea et al. trial [28] showed a reduction in the exacerbation rate (2.39 vs. 3.48 exacerbations/year, *p* = 0.034) and an improvement in quality of life (SGRQ −12.3 vs. −1.2, *p* = 0.028) with HFNT compared with usual care. Similarly, our results showed an improvement in the total SGRQ score and an even more striking difference in the exacerbation rate (2.6 vs. 4.5 exacerbations/year, *p* = 0.006) in the HFNT group, despite a high prevalence of chronic colonization with *Pseudomonas aeruginosa* (40%) in the analyzed cohort, which is an important driving factor for uncontrolled disease and frequent hospitalizations [56,57]. This is particularly relevant if we consider that BE exacerbations and hospitalizations may significantly increase patients’ morbidity and mortality and impair health-related quality of life [6,56,57,58] with a substantial burden on health systems, often due to a significant rise in antibiotics prescriptions and unscheduled visits [7]. Moreover, domiciliary HFNT has already been shown to represent a cost-effective intervention in patients with COPD, decreasing hospital admissions [59]. Thus, considering the similar physio-pathological features of these two muco-obstructive diseases that frequently coexist and in light of our findings, it is likely to hypothesize that domiciliary HFNT could be a valuable non-pharmacological additional treatment in frequently exacerbating BE patients.

We believed that our remarkable preliminary results in terms of reduction in exacerbations in the HFNT group might be due to a prolonged HFNT treatment duration. We reported an average usage of 6.3 h per day which is more than three times higher than the one reported in the Rea et al. study (1.7 h per day) [24]. Previous reports showed that airway humidification lasting from 30 min to 3 h could facilitate postural drainage and lung clearance in patients with BE [30], raising the assumption that longer exposures to HFNT are most beneficial. Moreover, our results are in line with the average usage time reported in recent studies focused on COPD [25,26,27]. Interestingly, we found a linear relationship between the time of HFNT use and improvements in exacerbation and hospitalization rates and pulmonary function parameters.

Although our patients continued to report chronic mucus production, the phlegm characteristics have changed after long-term HFNT utilization, moving from mucopurulent and purulent to mucoid features. This shift in mucus characteristics is clinically relevant, as sputum color is associated with bacterial colonization and neutrophilic inflammation, characterized by NETosis, which contributes to further mucus stiffening [60] and can predict the risk of future exacerbations [37]. This change in sputum characteristics was combined with the improved ease of mucus expectoration, as demonstrated by a decrease in VAS of two points compared to the control group. These results are likely related to the capability of HFNT to deliver warm and humidified air, which can positively affect the mucociliary beating and transport [51,61,62] and mobilization of airways mucus, thus reducing secretions load and viscosity and facilitating sputum expectoration [63,64].

Moreover, we detected not only a significant change in FEV_1_% and FVC% between the groups but also in FEF_25–75_%. A possible explanation for these findings is the beneficial effect of HFNT in improving small airways resistance and peripheral lung reactance [65] and in reducing mucus plugs in the distal small airways, which are likely to be the primary cause of the obstructive defect seen in BE [66].

The major strengths of this observational study are the inclusion of patients from prospective ongoing databases, the careful clinical and radiological assessment of BE severity, and the intra- and inter groups evaluation of effectiveness. Moreover, we evaluated the effectiveness of domiciliary HFNT use also in terms of change in symptoms and exacerbations, which are considered good clinical markers of treatment response in clinical research on BE [67].

However, this study has several limitations. First, due to the bicentric retrospective observational design, the study might reflect our local practice limiting the external validity of the results. Second, the small sample size limits the generalizability of our findings. Third, the presence of some missing data, such as SGRQ domains, may have reduced the robustness of the analysis. Moreover, we cannot exclude that the COVID-19 pandemic might have affected the exacerbation rates in a limited number of patients. Finally, our follow-up is limited to 12 months; therefore, we cannot assess the outcomes trajectories. Future high-quality research is required to confirm these findings and provide further evidence of the clinical efficacy of home HFNT. A large clinical trial (NCT04102774) [68] aiming to assess the effect of long-term overnight use of HFNT in adults with BE and frequent exacerbations/year is currently ongoing.

## 5. Conclusions

Long-term domiciliary use of HFNT as an add-on treatment in patients with BE may be beneficial in reducing exacerbations and hospitalizations and ameliorating symptoms and quality of life.

## Figures and Tables

**Table 1:** Baseline patients’ characteristics.

	HFNT (*n* = 20)	Control (*n* = 20)	*p*-Value
**Age, years, mean (SD)**	70.7 (9.4)	68.6 (8.9)	0.4835
**Female, *n* (%)**	14 (70)	14 (70)	0.9999
**BMI, Kg/m^2^, mean (SD)**	24.8 (7.6)	25.6 (7.9)	0.7542
**Smoking history, *n* (%)**			
Current	1 (5)	1 (5)	0.9999
Ex-smoker	4 (20)	6 (30)	0.7164
**Cardiovascular disease, *n* (%)**	12 (60)	11 (55)	0.9999
**GERD, *n* (%)**	13 (65)	11 (55)	0.7475
**Depression and/or anxiety, *n* (%)**	7 (35)	5 (25)	0.7311
**Bronchiectasis aetiology, *n* (%)**			
Idiopathic	4 (20)	6 (30)	0.7164
Post-infective	7 (35)	4 (20)	0.4801
Chronic obstructive pulmonary disease	5 (25)	5 (25)	0.9999
Immunodeficiency	1 (5)	4 (20)	0.3416
Primary ciliary dyskinesia	1 (5)	1 (5)	0.9999
Inflammatory bowel disease	1 (5)	n/a	n/a
Severe asthma	2 (10)	2 (10)	0.9999
**ICS-LABA, *n* (%)**	5 (25)	5 (25)	0.9999
**ICS-LABA and/or LAMA, *n* (%)**	10 (50)	10 (50)	0.9999
**LAMA + LABA, *n* (%)**	5 (25)	5 (25)	0.9999
**Long-term macrolide therapy, *n* (%)**	10 (50)	7 (32.5)	0.5231
**Airway clearance and/or pulmonary rehabilitation, *n* (%)**	20 (100)	20 (100)	0.9999
**Annual exacerbations, mean (SD)**	4.6 (1.4)	4 (1.4)	0.5964
**Annual severe exacerbations requiring hospitalizations, mean (SD)**	1.6 (0.6)	1.4 (0.6)	0.8091
**mMRC dyspnea scale, mean (SD)**	2.6 (0.6)	2.4 (0.7) ^†^	0.3398
**Difficulty of mucus expectoration VAS, mean (SD)**	6.9 (2)	7.1 (1.7) ^†^	0.4745
**Mucus features, *n* (%)**			
Mucous	2 (10)	1 (9.1) ^†^	0.9999
Mucopurulent	10 (50)	7 (63.6) ^†^	0.7074
Purulent	8 (40)	3 (27.3) ^†^	0.6979
**SGRQ, mean (SD)**	65.1 (14.6)	69.6 (13.3) ^†^	0.4055
**SpO_2_ at rest, %, mean (SD)**	92.8 (1.9)	93.3 (1.9) ^†^	0.6959
**Patients with SpO_2_ at rest <92% at room air, *n* (%)**	5 (25)	4 (20)	0.9999
**Long-term oxygen therapy, *n* (%)**	4 (20)	4 (20)	0.9999
**FEV_1_, % predicted, mean (SD)**	58.8 (18)	63.2 (17.7) ^¶^	0.4652
**FVC, % predicted, mean (SD)**	69.2 (12.6)	76.7 (17) ^¶^	0.1489
**FEF_25–75_, % predicted, mean (SD)**	45.2 (12.9)	46.4 (12.8) ^†^	0.9907
**Alpha-1 antitrypsin, mg/dL, median (IQR)**	159 (149–182)	162 (151–190)	0.6061

^†^ Data from 11 patients; ^¶^ data from 17 patients. Abbreviations: BMI, body mass index; GERD, gastroesophageal reflux disease; HFNT, high flow nasal therapy; ICS, inhaled corticosteroids; LABA, long-acting beta-agonist; LAMA, long-acting muscarinic antagonist; LTOT, long term oxygen therapy; mMRC, modified medical research council; VAS, visual analog scale; SGRQ, Saint George Respiratory Questionnaire; SpO_2_, peripheral blood oxygen saturation; FEV_1_, forced expiratory volume in the first second; FVC, forced vital capacity; FEF_25–75_, forced expiratory flow between 25% and 75% of FVC.

**Table 2:** Bronchiectasis severity assessment and microbial sputum cultures.

	HFNT (*n* = 20)	Control (*n* = 20)	*p*-Value
**Bhalla score, mean (SD)**	10.1 (3.5)	13.3 (3.7)	0.0344
**Bhalla, *n* (%)**			
Mild (16–25)	1 (5)	3 (27.3)	0.1154
Moderate (9–15)	13 (65)	7 (63.6)	0.9999
Severe (0–8)	6 (30)	1 (9.1)	0.3717
**BSI, mean (SD)**	12.4 (3.6)	12.3 (3.5)	0.8950
**BSI, *n* (%)**			
Mild (0–4)	0 (0)	0 (0)	0.9999
Moderate (5–8)	2 (10)	2 (10)	0.9999
Severe (≥9)	18 (90)	18 (90)	0.9999
**Sputum cultures, *n* (%)**			
Negative	6 (30)	5 (25)	0.9999
*Pseudomonas aeruginosa*	8 (40)	9 (45)	0.9999
Other bacteria	3 (7.5)	3 (7.5)	0.9999
*Aspergillus fumigatus*	1 (5)	1 (5)	0.9999

Bold entry highlights statistically significant *p*-value. Abbreviations: BSI, bronchiectasis severity index; COPD, Chronic obstructive pulmonary disease; HFNT, high-flow nasal therapy.

**Table 3:** Outcomes after 12 months.

	HFNT		Control			
	12 Months	Change from Baseline *	12 Months	Change from Baseline *	Difference * and Relative Risk	*p*-Value
**Annual exacerbations, mean (SD)**	2.6 (1.4)	−2 (−2.9 to −1.1)	3.9 (1.6)	−0.1 (−0.9 to 0.7)	−1.9 (−2.8 to −0.9)	**0.0005**
**Patients with ≥ 3 annual exacerbations, *n* (%)**	12 (60)	−7 (−35)	17 (85)	−0 (−0)	0.6 (0.4 to 0.0)	**0.0084**
**Annual severe exacerbations requiring hospitalizations, mean (SD)**	0.7 (0.5)	−0.9 (−1.1 to −0.7)	1.2 (0.8)	−0.2 (−0.5 to 0.1)	−0.7 (−1.1 to −0.3)	**0.0006**
**Patients with ≥ 1 annual hospitalization, *n* (%)**	13 (65)	−7 (−35)	16 (80)	−4 (−20)	0.8 (0.5 to 1.2)	0.4801
**mMRC dyspnea scale, mean (SD)**	2 (0.7)	−0.6 (−1.0 to −0.3)	2.3 (0.5)	−0.1 (−0.6 to +0.4) ^†^	−0.5 (−0.0 to +1.2)	0.6447
**Difficulty of mucus expectoration VAS, mean (SD)**	4.9 (2.2)	−2 (−2.7 to −1.3)	7.3 (2.1)	+0.2 (−1.9 to +2.2) ^†^	−2.2 (−3.9 to −0.5)	**0.0124**
**Mucus features, *n* (%)**						
Mucous	9 (45)	+7 (+35)	2 (18.9)	+1 (8.9)	-	0.2409
Mucopurulent	6 (30)	−4 (−20)	5 (45.5)	−2 (45.5)	-	0.4524
Purulent	5 (25)	−3 (−15)	4 (36.4)	+1 (36.4)	-	0.6828
**SGRQ, mean (SD)**	55.2 (11.3)	−9.9 (−12.9 to −6.8)	70 (10.5)	+0.5 (−9.0 to +10.6) ^†^	−10.4 (−20.2 to −0.6)	**0.0391**
**SpO_2_ at rest, %, mean (SD)**	93.8 (1.7)	+1 (+0.3 to +1.6)	93.4 (1.9)	+0 (−1 to +1) ^†^	+1 (−0.1 to +2)	0.0845
**FEV_1_, % predicted, mean (SD)**	64.3 (18.7)	+5.6 (+3.5 to +7.6)	62.7 (17.8)	−0.6 (−5.4 to +4.2) ^¶^	+6.1 (+1 to +11.3)	**0.0219**
**FVC, % predicted, mean (SD)**	72.8 (11.6)	+3.6 (+2.3 to +5.0)	75.8 (18.9)	−1 (−4.7 to +2.8) ^¶^	+4.6 (+0.8 to +8.3)	**0.0188**
**FEF_25–75_, % predicted, mean (SD)**	55.1 (12.2)	+9.9 (+8.3 to +11.5)	42.9 (11.5)	−3.5 (−5.6 to −1.5) ^†^	+13.4 (+11 to +15.9)	**<0.0001**

Bold entries highlight statistically significant *p*-values. * Values are expressed as means with 95% confidence interval for continuous data and as numbers (*n*) and percentages (%) for categorical variables. ^†^ Data from 11 patients; ^¶^ data from 17 patients. Abbreviations: mMRC, modified medical research council; VAS, visual analog scale; SGRQ, Saint George Respiratory Questionnaire; SpO_2_, peripheral blood oxygen saturation; FEV_1_, forced expiratory volume in the first second; FVC, forced vital capacity; FEF_25–75_, forced expiratory flow between 25% and 75% of FVC.

## Data Availability

The data presented in this study are available on reasonable request from the corresponding author.

## Abbreviations

BE	Bronchiectasis
BMI	Body mass index
BSI	Bronchiectasis Severity Index
COPD	Chronic obstructive pulmonary disease
FEF_25–75_	Forced expiratory flow between 25% and 75% of FVC
FEV_1_	Forced expiratory volume in the 1st second
FVC	Forced vital capacity
HFNT	High-flow nasal therapy
ICS	Inhaled corticosteroids
LABA	Long-acting beta2-agonist
LAMA	Long-acting muscarinic antagonist
mMRC	modified Medical Research Council
SGRQ	Saint George Respiratory Questionnaire
SpO_2_	Peripheral blood oxygen saturation
VAS	Visual analog scale
